# Prognostic values of *aquaporins* mRNA expression in human ovarian cancer

**DOI:** 10.1042/BSR20180108

**Published:** 2018-04-27

**Authors:** Mandika Chetry, Saisai Li, Hailing Liu, Xiaoli Hu, Xueqiong Zhu

**Affiliations:** Department of Obstetrics and Gynecology, The Second Affiliated Hospital of Wenzhou Medical University, Wenzhou 325027, China

**Keywords:** Aquaporins, ovarian cancer, Prognosis

## Abstract

Aquaporins (AQPs), a family of transmembrane channel, are composed of 13 identified members (AQP0–12). Accumulating evidences reported that AQPs were correlated with various biological roles and represented a prognostic predictor in various cancer types. However, the prognostic value of AQPs expression in ovarian cancer remains unclear. Using ‘Kaplan–Meier plotter’ (KM plotter) online database, we explored the predictive prognostic value of individual AQPs members’ mRNA expression to overall survival (OS) in different clinical data, such as histology, pathological grades, clinical stages, TP53 status, and applied chemotherapy in ovarian cancer patients. Our results revealed that higher *AQP0, AQP1*, and *AQP4* mRNA expression were correlated with poor OS, whereas higher AQP3, AQP5, AQP6, AQP8, AQP10, and AQP11 showed better OS in ovarian cancer patients. Moreover, AQP4 and AQP8 showed poor OS in TP53-mutated ovarian cancer patients and AQP1 presented unfavorable OS in both TP53 mutated and wild ovarian cancer patients. Additionally, *AQP3, AQP6*, and *AQP11* mRNA expression were correlated with better OS, whereas AQP0 and AQP1 showed poor OS in all ovarian cancer patients treated with Platin, Taxol, and Taxol + Platin chemotherapy. AQP5, AQP8, and AQP10 were associated with improved OS, however, AQP4 predicted unfavorable OS in all patients treated with Platin chemotherapy. Our results suggest that individual AQPs, except AQP2 and AQP9, are associated with unique prognostic significance and may thus act as new predictive prognostic indicators and potential drug therapeutic target in ovarian cancer.

## Introduction

Ovarian cancer contributes to most of the deaths from all gynecologic malignancies. It is the fifth leading cause of female health problems and deaths all over the world due to cancer [1]. Mostly ovarian cancer patients are diagnosed at an advanced stage; in recent years combined treatment of debulking surgery and chemotherapy have yielded a modest improvement in survival as a standard therapy [2]. Ovarian cancers predominantly have epithelial origins and are further classified into histological groups such as serous, clear cell, endometrioid, mucinous, transitional cell tumors, carcinosarcoma, undifferentiated carcinoma, mixed epithelial tumor, and so on [3]. Although considerable advances are present in early detection, chemotherapy, radical cure surgery, and targetted therapeutic management, many cases still (approximately 85%) recur [4] and develop gradual treatment resistance with lower 5-year survival rate (30%) [5]. Therefore, early establishment of a novel prognostic marker in ovarian cancer is the urgent requirement for the better prognosis and to improve clinical outcomes of these patients.

Aquaporins (AQPs) are a family of integral membrane proteins, which regulate the selective transport of water and other ions across membranes in response to osmotic or pressure gradient [6]. So far, 13 different types of AQPs have been identified. AQP0, AQP1, AQP2, AQP4, AQP5, AQP6, and AQP8 are mainly water-selective permeable to anions and ammonia [7,8], whereas AQP3, AQP7, AQP9, AQP10, and AQP12 also transport glycerol and possibly other small solutes, and AQP11 is not clear even as a water channel due to its unusual location in the cell [9,10]. Apart from its classical role as osmotic transepithelial and transcellular water regulators, AQPs are also involved in carcinogenesis of multiple cancers leading to tumor angiogenesis, cell migration, tumor progression, and tumor growth [9,11,12]. In particular, combined evidences have reported that AQPs could be used as an independent cancer prognostic factor and represent a potential target for different cancer therapy [13–15]. However, the prognostic values of *AQPs* mRNA expression in ovarian cancer have not been determined. In the present study, to demonstrate whether individual AQP genes are involved in prognostic significance of human ovarian cancer patients including various clinicopathological features such as pathological grade, clinical stage, TP53 status, and treatment strategy, we comprehensively explored data by using the Kaplan–Meier plotter (KM plotter).

## Materials and methods

An online KM plotter (http://kmplot.com/analysis) [16] database was utilized to evaluate relevant searches of individual AQPs members’ mRNA expression to overall survival (OS) of ovarian cancer patients. Recently, KM plotter is established with the potential access to 54675 genes that have been identified and validated in breast cancer [16–18], ovarian cancer [19,20], lung cancer [21], and gastric cancer [22]. In this study, 1816 ovarian cancer patients gene expressions and data on prognostic roles were established from Gene Expression Omnibus, Cancer Biomedical Informatics Grid, and The Cancer Genome Atlas cancer datasets [20]. Moreover, they provided the clinical data including stage, grade, histology, TP53 mutation status, and treatment of ovarian cancer patients. In the present study, from the available data source, we collected clinical outcomes such as pathological grades, clinical stages, TP53 status and chemotherapeutic strategy. In short, 11 AQP submembers (AQP0, AQP1, AQP2, AQP3, AQP4, AQP5, AQP6/2L, AQP8, AQP9, AQP10, and AQP11) were entered into the database (http://kmplot.com/analysis/index.php?p=service&cancer=ovar) to achieve Kaplan–Meier survival plots. Hazard ratio (HR) and 95% confidence intervals as well as log-rank *P* were calculated and displayed on the web page. A *P*-value of <0.05 was regarded as statistically significant.

## Results

**Figure 1:**
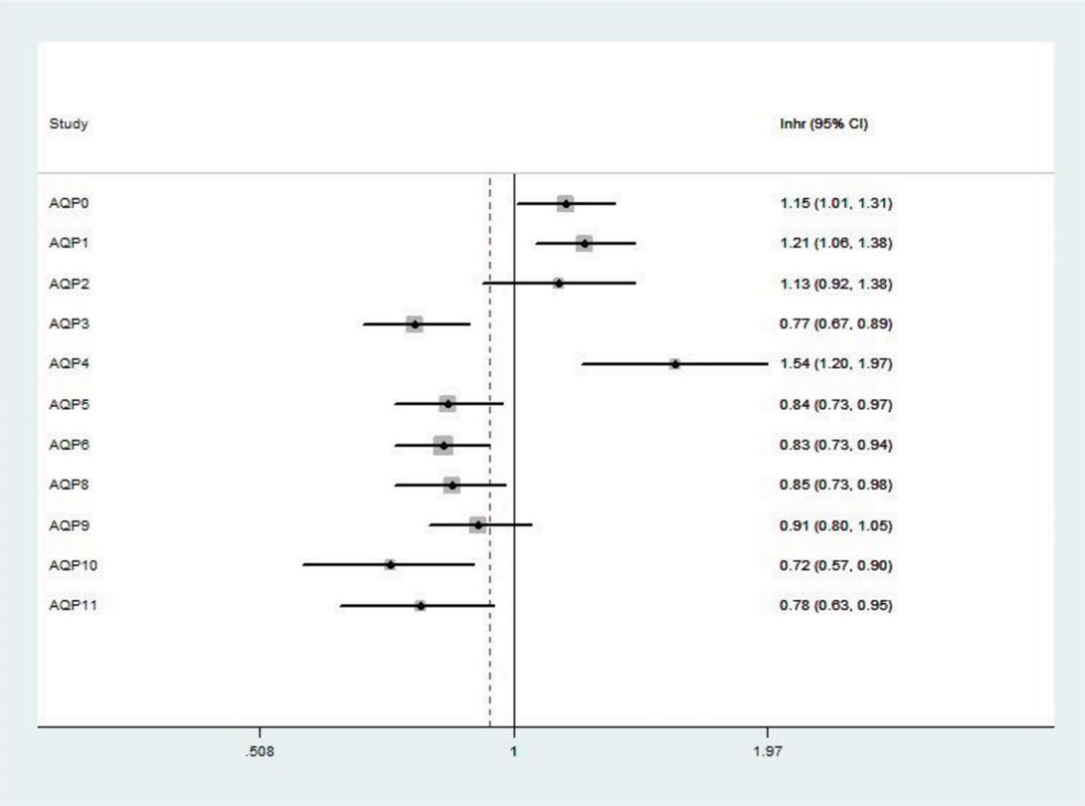
The prognostic HRs value of individual AQPs members in all ovarian cancer in www.kmplot.com

**Figure 2:**
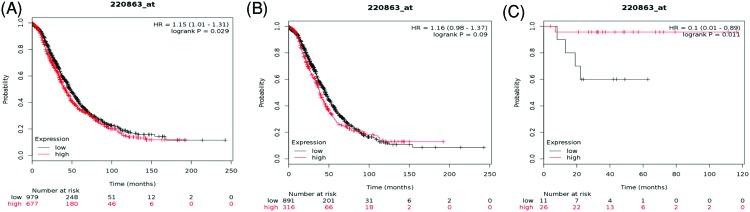
The prognostic value of AQP0 expression in ovarian cancer The prognostic value of AQP0 expression in www.kmplot.com. Affymetrix ID for AQP0: 220863_at. OS curves were plotted for (**A**) all the patients (*n*=1656), (**B**) serous cancer patients (*n*=1207), and (**C**) endometrioid cancer patients (*n*=37).

Amongst 13 different subtypes of AQPs, only data on 11 members were pooled in www.kmplot.com (Figure 1). Initially, the prognostic value of AQP0 was accessed in the database. For AQP0, Affymetrix ID is 220863_at. OS curves were plotted for all ovarian cancer patients (*n*=1656) (Figure 2A), for serous cancer patients (*n*=1207) (Figure 2B) and endometrioid cancer patients (*n*=37) (Figure 2C). Elevated expression of *AQP0* mRNA was significantly associated with poor OS in all ovarian cancer patients, HR = 1.15 (1.01–1.31), *P*=0.029. However, high expression of *AQP0* mRNA in endometrioid ovarian cancer patients displayed better OS, HR = 0.1 (0.01–0.89), *P*=0.011, while in serous ovarian cancer patients showed no any correlation with OS, HR = 1.16 (0.98–1.37), *P*=0.09.

**Figure 3:**
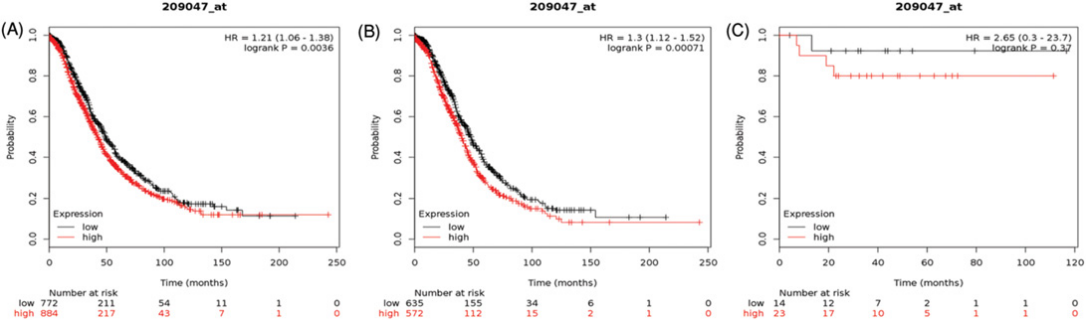
The prognostic value of AQP1 expression in ovarian cancer The prognostic value of AQP1 expression in www.kmplot.com. Affymetrix ID for AQP1: 209047_at. OS curves were plotted for (**A**) all the patients (*n*=1656), (**B**) serous cancer patients (*n*=1207), and (**C**) endometrioid cancer patients (*n*=37).

Next, the prognostic significance of *AQP1* mRNA expression was evaluated in the database. The desired Affymetrix ID is 209047_at. *AQP1* mRNA expression level revealed a remarkable correlation with worse OS amongst all the ovarian cancer patients, HR = 1.21 (1.06–1.38), *P*=0.0036 (Figure 3A). The histological subtype results showed that increased *AQP1* mRNA expression highly associated with unfavorable OS in patients of serous ovarian cancer, HR = 1.3 (1.12–1.52), *P*=0.00071 (Figure 3B), whereas expression level of *AQP1* mRNA in endometrioid cancer patient did not show any correlation with OS, HR = 2.65 (0.3–23.7), *P*=0.37 (Figure 3C).

**Figure 4:**
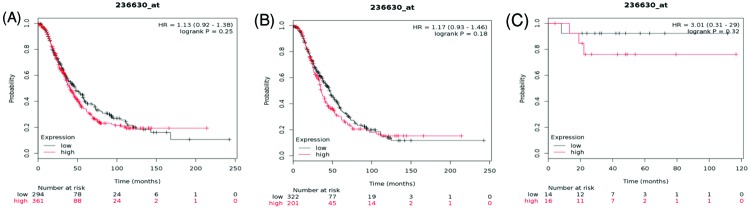
The prognostic value of AQP2 expression in ovarian cancer The prognostic value of AQP2 expression in www.kmplot.com. Affymetrix ID for AQP2: 236630_at. OS curves were plotted for (**A**) all the patients (*n*=655), (**B**) serous cancer patients (*n*=523), and (**C**) endometrioid cancer patients (*n*=30).

Similarly, for AQP2 Affymetrix ID is 236630_at. *AQP2* mRNA expression exhibited a null correlation with OS within all ovarian cancer patients as well as in serous and endometrioid histological subgroups, HR = 1.13 (0.92–1.38), *P*=0.25 (Figure 4A), HR = 1.17 (0.93–1.46), *P*=0.18 (Figure 4B) and HR = 3.01 (0.31–29), *P*=0.32 (Figure 4C), respectively.

**Figure 5:**
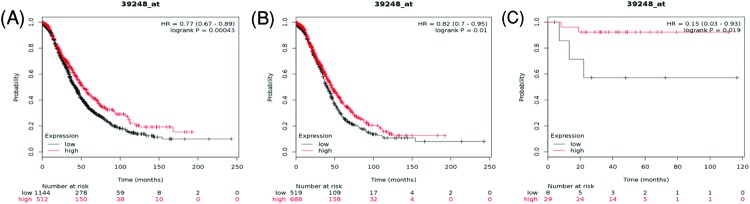
The prognostic value of AQP3 expression in ovarian cancer The prognostic value of AQP3 expression in www.kmplot.com. Affymetrix ID for AQP3: 39248_at. OS curves were plotted for (**A**) all the patients (*n*=1656), (**B**) serous cancer patients (*n*=1207), and (**C**) endometrioid cancer patients (*n*=37).

Then the prognostic value of AQP3 was further determined in the database. The Affymetrix ID is 39248_at. *AQP3* mRNA expression was significantly correlated with better OS for all ovarian cancer patients, HR = 0.77 (0.67–0.89), *P*=0.00043 (Figure 5A), serous ovarian cancer patients, HR = 0.82 (0.7–0.95), *P*=0.01 (Figure 5B), and endometrioid ovarian cancer patients, HR = 0.15 (0.03–0.93), *P*=0.019 (Figure 5C).

**Figure 6:**
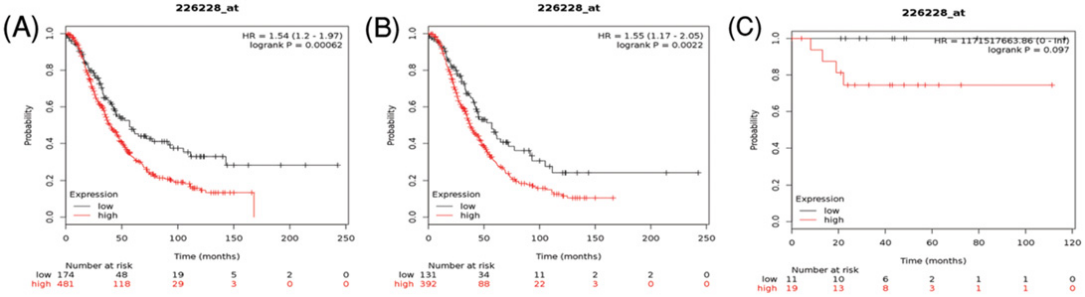
The prognostic value of AQP4 expression in ovarian cancer The prognostic value of AQP4 expression in www.kmplot.com. Affymetrix ID for AQP4: 226228_at. OS curves were plotted for (**A**) all the patients (*n*=655), (**B**) serous cancer patients (*n*=523), and (**C**) endometrioid cancer patients (*n*=30).

Figure 6 demonstrated the prognostic value of AQP4 in the database. The Affymetrix ID for AQP4: 226228_at. Increased expression of *AQP4* mRNA was significantly correlated with poor OS for all ovarian cancer, HR = 1.54 (1.2–1.97), *P*=0.00062 (Figure 6A) and serous ovarian cancer, HR = 1.55 (1.17–2.05), *P*=0.0022 (Figure 6B). However, overexpression of *AQP4* mRNA for endometrioid ovarian cancer was not correlated with OS, HR = 1171517663.86 (0–inf), *P*=0.097 (Figure 6C).

**Figure 7:**
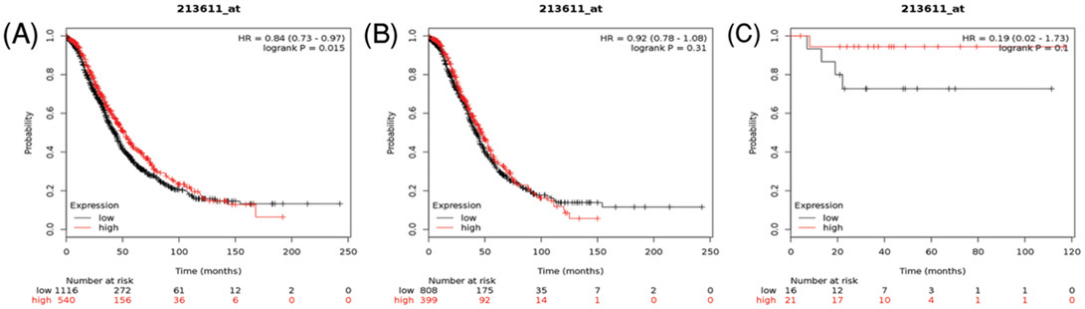
The prognostic value of AQP5 expression in ovarian cancer The prognostic value of AQP5 expression in www.kmplot.com. Affymetrix ID for AQP5: 213611_at. OS curves were plotted for (**A**) all the patients (*n*=1656), (**B**) serous cancer patients (*n*=1207), and (**C**) endometrioid cancer patients (*n*=37).

Figure 7 illustrated the prognostic value of AQP5 in the database. Affymetrix IDs for AQP5: 213611_at. The higher expression of *AQP5* mRNA level was associated with better OS in all ovarian cancer patients, HR = 0.84 (0.73–0.97), *P*=0.015 (Figure 7A). Nevertheless, *AQP5* mRNA expression both in serous ovarian cancer and endometrioid ovarian cancer did not show any correlation with OS, HR = 0.92 (0.78–1.08), *P*=0.31 (Figure 7B), and HR = 0.19 (0.02–1.73), *P*=0.1 (Figure 7C), respectively.

**Figure 8:**
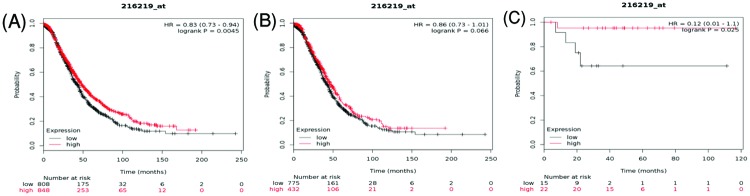
The prognostic value of AQP6 expression in ovarian cancer The prognostic value of AQP6 expression in www.kmplot.com. Affymetrix ID for AQP6: 216219_at. OS curves were plotted for (**A**) all the patients (*n*=1656), (**B**) serous cancer patients (*n*=1207), and (**C**) endometrioid cancer patients (*n*=37).

AQP6 is also known as AQP2L. Figure 8 showed the prognostic significance of AQP6 in the database. Affymetrix ID for AQP6/AQP2L is 216219_at. The elevated expression of *AQP6/AQP2L* mRNA was associated with favorable OS for all ovarian cancer patients and endometrioid ovarian cancer patients, HR = 0.83 (0.73–0.94), *P*=0.0045 (Figure 8A), HR = 0.12 (0.01–1.1), *P*=0.025 (Figure 8C), respectively. While outcome measures regarding serous ovarian cancer, *AQP6/AQP2L* mRNA showed no any correlation with OS, HR = 0.86 (0.73–1.01), *P*=0.066 (Figure 8B).

**Figure 9:**
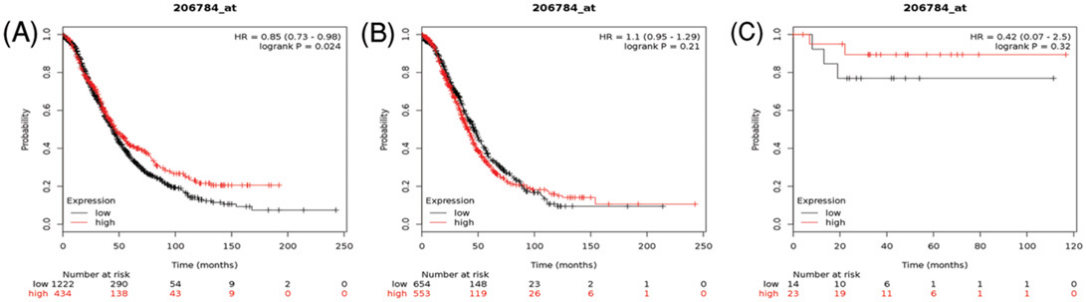
The prognostic value of AQP8 expression in ovarian cancer The prognostic value of AQP8 expression in www.kmplot.com. Affymetrix ID for AQP8: 206784_at. OS curves were plotted for (**A**) all the patients (*n*=1656), (**B**) serous cancer patients (*n*=1207), and (**C**) endometrioid cancer patients (*n*=37).

Figure 9 presented the prognostic value of *AQP8* mRNA expression in the database. Affymetrix ID for AQP8 was 206784_at. Overexpression of AQP8 was significantly correlated with favorable OS in all ovarian cancer patients, HR = 0.85 (0.73–0.98), *P*=0.024 (Figure 9A). However, it did not show any correlation with OS in serous and endometrioid ovarian cancer patients, HR = 1.1 (0.95–1.29), *P*=0.21 (Figure 9B), HR = 0.42 (0.07–2.5), *P*=0.32 (Figure 9C), respectively.

**Figure 10:**
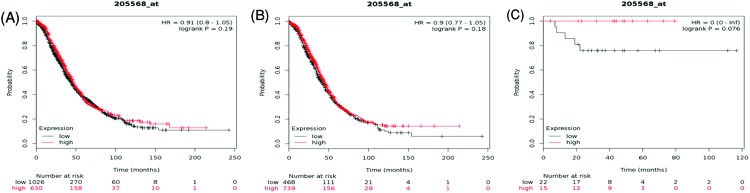
The prognostic value of AQP9 expression in ovarian cancer The prognostic value of AQP9 expression in www.kmplot.com. Affymetrix ID for AQP9: 205568_at. OS curves were plotted for (**A**) all the patients (*n*=1656), (**B**) serous cancer patients (*n*=1207), and (**C**) endometrioid cancer patients (*n*=37).

Figure 10 revealed the prognostic value of *AQP9* mRNA expression in the database. Affymetrix ID for AQP9 is 205568_at. Higher expression of AQP9 was neither correlated with OS for all ovarian cancer HR = 0.91 (0.8–1.05), *P*=0.19 (Figure 10A) nor with serous, HR = 0.9 (0.77–1.05), *P*=0.18 (Figure 10B) and endometrioid cancer, HR = 0 (0–inf), *P*=0.076 (Figure 10C).

**Figure 11:**
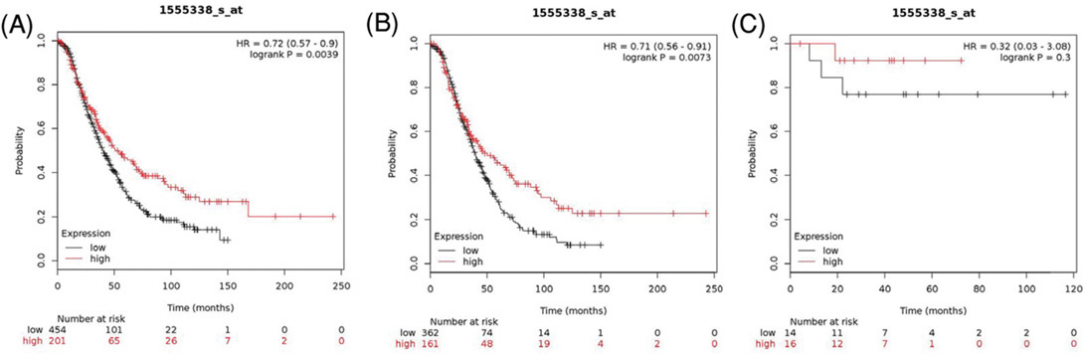
The prognostic value of AQP10 expression in ovarian cancer The prognostic value of AQP10 expression in www.kmplot.com. Affymetrix ID for AQP10: 1555338_s_at. OS curves were plotted for (**A**) all the patients (*n*=655), (**B**) serous cancer patients (*n*=523), and (**C**) endometrioid cancer patients (*n*=30).

Figure 11 showed the prognostic value of *AQP10* mRNA expression in the database. Affymetrix ID for AQP10 was 1555338_s_at. AQP10’s high mRNA expression was found to be associated with better OS for all ovarian cancer patients, HR = 0.72 (0.57–0.9), *P*=0.0039 (Figure 11A). Furthermore, high *AQP10* mRNA expression was also correlated with better OS for serous ovarian cancer patients, HR = 0.71 (0.56–0.91), *P*=0.0073 (Figure 11B). However, the expression level of AQP10 was not correlated with endometrioid ovarian cancer, HR = 0.32 (0.03–3.08), *P*=0.3 (Figure 11C).

**Figure 12:**
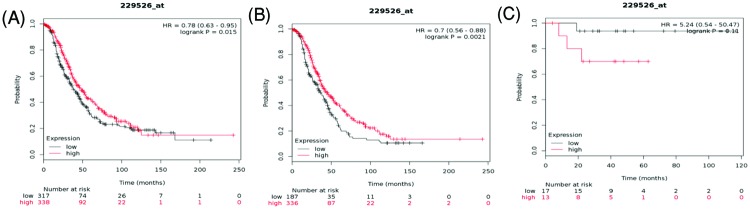
The prognostic value of AQP11 expression in ovarian cancer The prognostic value of AQP11 expression in www.kmplot.com. Affymetrix ID for AQP11: 229526_at. OS curves were plotted for (**A**) all the patients (*n*=655), (**B**) serous cancer patients (*n*=523), and (**C**) endometrioid cancer patients (*n*=30).

Finally, we assessed the prognostic significance of *AQP11* mRNA expression in the database. Affymetrix IDs for AQP11 was 229526_at. High level of *AQP11* mRNA was significantly associated with better OS for all ovarian cancer patients, HR = 0.78 (0.63–0.95), *P*=0.015 (Figure 12A) and serous ovarian cancer, HR = 0.7 (0.56–0.88), *P*=0.0021 (Figure 12B). However, increased *AQP11* mRNA in endometrioid ovarian cancer was not associated with OS, HR = 5.24 (0.54–50.47), *P*=0.11 (Figure 12C).

In the present study, constitutive expression of *AQP2* and *AQP9* mRNA showed not any correlation to the prognosis of all ovarian cancer patients, serous ovarian cancer patients, as well as endometrioid ovarian cancer patients. Therefore, the associations of the other nine AQP submembers mRNA with other clinicopathological features were further examined, including the pathological grades (Table 1), clinical stages (Table 2), TP53 mutation (Table 3) and chemotherapy agents (Table 4) of ovarian cancer patients. As shown in the Table 1, overexpression of AQP3, AQP6/2L, and AQP11 were associated with favorable OS in grade III ovarian cancer patients. Subsequent expression of *AQP0, AQP5*, and *AQP10* mRNA expression had better OS in grade I ovarian cancer patients. On the other hand, mRNA expression of AQP1 (grades I and II), AQP4 (grades I, II, and III), AQP6/2L (grade I), AQP10 (grade II) showed a worse OS in ovarian cancer patients. Whereas, *AQP8* mRNA expression showed not any correlation with all pathological grade of ovarian cancer.

**Table 1:** Correlation of AQPs gene expression level with OS in different pathological grades in ovarian cancer patients

AQPs	Pathological grade	Cases	HR (95% CI)	*P*-value
AQP0	I	56	0.26 (0.09–0.75)	0.0076*
	II	324	1.2 (0.88–1.63)	0.25
	III	1015	1.1 (0.93–1.31)	0.26
AQP1	I	56	2.77 (0.99–7.76)	0.044*
	II	324	1.49 (1.1– 2.01)	0.0094*
	III	1015	1.15 (0.97–1.36)	0.12
AQP3	I	56	0.4 (0.14–1.12)	0.07
	II	324	0.79 (0.56–1.11)	0.17
	III	1015	0.81 (0.68–0.95)	0.01*
AQP4	I	41	5.21 (1.43–18.97)	0.0055*
	II	162	2.01 (1.26–3.22)	0.0028*
	III	392	1.49 (1.1–2.02)	0.0093*
AQP5	I	56	0.37 (0.14–0.99)	0.04*
	II	324	1.27 (0.93–1.72)	0.13
	III	1015	0.91 (0.77–1.08)	0.29
AQP6/AQP2L	I	56	2.68 (1.02–7.05)	0.038*
	II	324	0.77 (0.57–1.05)	0.093
	III	1015	0.83 (0.71–0.99)	0.034*
AQP8	I	56	0.34 (0.1–1.18)	0.075
	II	324	0.77 (0.56–1.06)	0.11
	III	1015	0.85 (0.7–1.02)	0.081
AQP10	I	41	0.14 (0.04–0.46)	0.00021*
	II	162	1.67 (1.04–2.67)	0.031*
	III	392	0.76 (0.57–1)	0.051
AQP11	I	41	2.12 (0.66–6.79)	0.19
	II	162	0.66 (0.42–1.02)	0.057
	III	392	0.7 (0.55–0.91)	0.0067*

* *P*<0.05.

**Table 2:** Correlation of AQPs gene expression level with OS in different clinical stages in ovarian cancer patients

AQPs	Clinical stages	Cases	HR (95% CI)	*P*-value
AQP0	I + II	135	0.49 (0.21–1.13)	0.088
	III + IV	1220	0.92 (0.78–1.09)	0.34
AQP1	I + II	135	2.16 (0.98–4.77)	0.05*
	III + IV	1220	1.19 (1.02–1.39)	0.027*
AQP3	I + II	135	0.62 (0.27–1.43)	0.26
	III + IV	1220	0.82 (0.69–0.97)	0.017*
AQP4	I + II	83	5.97 (0.78–45.49)	0.05*
	III + IV	487	1.39 (1.09–1.77)	0.0072*
AQP5	I + II	135	6.33 (1.48–27.11)	0.0047*
	III + IV	1220	0.85 (0.72–1)	0.054
AQP6/AQP2L	I + II	135	0.43 (0.19–0.95)	0.032*
	III + IV	1220	0.75 (0.65–0.88)	0.00024*
AQP8	I + II	135	0.52 (0.24–1.14)	0.096
	III + IV	1220	0.77 (0.65–0.91)	0.0024*
AQP10	I + II	83	0.37 (0.12–1.17)	0.079
	III + IV	487	0.69 (0.53–0.89)	0.0041*
AQP11	I + II	83	0.56 (0.16–1.97)	0.36
	III + IV	487	0.84 (0.67–1.05)	0.12

* *P*<0.05.

**Table 3:** Correlation of AQPs genes expression with OS in ovarian cancer patients with TP53 mutation status

AQPs	TP53 mutation	Cases	HR (95% CI)	*P*-value
AQP0	Mutant	506	1.16 (0.91–1.49)	0.22
	Wild	94	1.55 (0.9–2.67)	0.11
AQP1	Mutant	506	1.38 (1.1–1.73)	0.0051*
	Wild	94	1.87 (1.03–3.4)	0.036*
AQP3	Mutant	506	1.16 (0.93–1.47)	0.19
	Wild	94	1.39 (0.8–2.4)	0.24
AQP4	Mutant	124	1.75 (1.18–2.59)	0.0045*
	Wild	19	Not available	Not available
AQP5	Mutant	506	0.82 (0.65–1.04)	0.1
	Wild	94	0.76 (0.42–1.39)	0.38
AQP6/AQP2L	Mutant	506	0.84 (0.65–1.07)	0.16
	Wild	94	0.56 (0.3–1.02)	0.056
AQP8	Mutant	506	1.33 (1.06–1.67)	0.013*
	Wild	94	1.41 (0.82–2.42)	0.21
AQP10	Mutant	124	0.74 (0.49–1.12)	0.16
	Wild	19	Not available	Not available
AQP11	Mutant	124	1.31 (0.9–1.91)	0.16
	Wild	19	Not available	Not available

* *P*<0.05.

**Table 4:** Correlation of AQPs genes expression level with OS in ovarian cancer patients with different chemotherapeutic agents

AQPs	Chemotherapy	Cases	HR (95% CI)	*P*-value
AQP0	Contains Platin	1409	1.16 (1.01–1.34)	0.033*
	Contains Taxol	793	1.24 (1.03–1.5)	0.025*
	Contains Taxol + Platin	776	1.28 (1.06–1.55)	0.012*
AQP1	Contains Platin	1409	1.28 (1.09–1.49)	0.0022*
	Contains Taxol	793	1.25 (1.03–1.52)	0.026*
	Contains Taxol + Platin	776	1.26 (1.03–1.54)	0.021*
AQP3	Contains Platin	1409	0.82 (0.7–0.96)	0.014*
	Contains Taxol	793	0.76 (0.61–0.95)	0.016*
	Contains Taxol + Platin	776	0.77 (0.62–0.96)	0.018*
AQP4	Contains Platin	478	1.52 (1.13–2.03)	0.005*
	Contains Taxol	357	1.31 (0.98–1.74)	0.064
	Contains Taxol + Platin	356	1.32 (0.99–1.75)	0.06
AQP5	Contains Platin	1409	0.85 (0.73–0.99)	0.033*
	Contains Taxol	793	1.15 (0.95–1.39)	0.14
	Contains Taxol + Platin	776	1.17 (0.96–1.41)	0.12
AQP6/AQP2L	Contains Platin	1409	0.8 (0.7–0.93)	0.0023*
	Contains Taxol	793	0.79 (0.65–0.95)	0.013*
	Contains Taxol + Platin	776	0.77 (0.63–0.94)	0.012*
AQP8	Contains Platin	1409	0.86 (0.74–0.99)	0.038*
	Contains Taxol	793	0.84 (0.69–1.03)	0.096
	Contains Taxol + Platin	776	0.83 (0.68–1.02)	0.07
AQP10	Contains Platin	478	0.74 (0.57–0.97)	0.027*
	Contains Taxol	357	1.32 (0.92–1.89)	0.14
	Contains Taxol + Platin	356	1.3 (0.91–1.86)	0.15
AQP11	Contains Platin	478	0.74 (0.58–0.95)	0.017*
	Contains Taxol	357	0.65 (0.48–0.89)	0.0067*
	Contains Taxol + Platin	356	0.65 (0.47–0.88)	0.0057*

* *P*<0.05.

In addition, Table 2 showed the association between AQPs expression and clinical stages, in which *AQP3* (stage III + IV), *AQP8* (stage III + IV), *AQP10* (stage III + IV), and *AQP6/2L* (stages I + II and III + IV) mRNA expressions were significantly associated with favorable OS in ovarian cancer. Whereas AQP1 (stages I + II and III + IV), AQP4 (stages I + II and III + IV), and AQP5 (stage I + II) expressions revealed remarkably unfavorable OS in all ovarian cancer patients. However, AQP0 and AQP11 exhibited no correlation with any clinical stage of ovarian cancer.

While further assessing the prognostic significance between individual *AQPs* mRNA expression and TP53 mutation, AQP4 and AQP8 showed significantly poor OS in TP53-mutated ovarian cancer patients (Table 3), similarly AQP1 showed unfavorable OS in both TP53 mutated and wild ovarian cancer patients. However, AQP0, AQP3, AQP5, AQP6/2L, AQP10, and AQP11 revealed no correlation in both TP53 mutated and wild ovarian cancer patients.

Additionally, Table 4 showed that *AQP6/AQP2L, AQP3*, and *AQP11* mRNA expression were correlated with better OS in all ovarian cancer patients treated with Platin, Taxol, and Taxol + Platin chemotherapeutic agents. AQP10, AQP5, and AQP8 were associated with improved OS in all patients treated with Platin chemotherapy, whereas AQP0 and AQP1 mRNA expression was associated with poor OS in all patients treated with Platin, Taxol, and Taxol + Platin chemotherapy. On the other hand, AQP4 expression was associated with unfavorable OS in all patients treated with Platin chemotherapy.

## Discussion

In the present study, we comprehensively retrieved the prognostic significance of individual *AQPs* mRNA expression in ovarian cancer by using the KM plotter. Amongst the members of AQPs family, only *AQP2* and *AQP9* mRNA had no effect on the OS of ovarian cancers, i.e. *AQP0, AQP1*, and *AQP4* mRNA expression were associated with poor OS, whereas high expression of AQP3, AQP5, AQP6, AQP8, AQP10, and AQP11 were associated with better OS for ovarian cancer patients.

In a previous study, reduced expression of water channels AQP2 was found to be correlated with cisplatin-induced polyuria and end-organ resistance in rats [23]. But so far, the role of AQP2 in cancers has not yet been explored. In the present study, we explored the prognostic significance of AQP2 in 1816 ovarian cancer patients by using the KM plotter database. Our results showed that high expression of *AQP2* mRNA had no effect on the prognosis of all ovarian cancer patients, serous ovarian cancer patients, as well as in endometrioid ovarian cancer patients. As for AQP9, Huang et al. [24] reported that AQP9 could serve as an independent predictive biomarker for adjuvant chemotherapy, and they demonstrated that high AQP9 expression was related to better disease-free survival in colorectal cancer patients treated with chemotherapy. However, the study of Tan et al. [25] indicated that the expression of both *AQP9* mRNA and protein was significantly up-regulated in astrocytic tumors and was positively associated with pathological grade, suggesting that AQP9 might play a critical role in the malignant progression of brain astrocytic tumors. Yang et al. [26] demonstrated that AQP9 protein expression in malignant and borderline tumors was significantly higher than in benign tumor and normal ovarian tissue. And high AQP9 expression level was found to be positively associated with tumor grade and histological type, they therefore proposed that overexpression of AQP9 might represent an important factor in ovarian carcinogenesis. Nevertheless, little is known about the prognostic value of AQP9 in ovarian cancer patients. In the present study, our data revealed that *AQP9* mRNA expression exhibited a null correlation with the prognosis in different histological types of ovarian cancer patients. Thus, the prognostic value of AQP2 and AQP9 in ovarian cancer needs further exploration.

To our knowledge, there was no study about the role of either AQP0 protein or mRNA in ovarian cancer, we conducted the first report on the prognostic significance of AQP0 in ovarian cancer. In our study, we demonstrated that high expression of *AQP0* mRNA was significantly associated with poor OS in all ovarian cancer patients. Several previous studies reported that high AQP1 expression was related to an unfavorable prognosis including cutaneous melanoma, pharyngeal carcinomas, urothelial carcinoma, and breast cancer [27–30]. In ovarian cancer, Yang et al. [31] found that high expression of AQP1 might play a key role in ovarian carcinogenesis, progression, and ascites formation. However, the prognostic value of AQP1 in ovarian cancer has not yet been studied. In our current study, we discovered overexpression of *AQP1* mRNA was associated with poor OS for all ovarian cancer patients, especially for serous ovarian cancer, with well and moderate differentiation (pathological grades I and II) as well as in all clinical stages (I–IV). As for AQP4, Sun et al. [32] revealed that AQP4 modulated not only water and ion homeostasis but also regulated the mechanism of ovarian hormone and neurotransmitter. Of note, earlier studies demonstrated that high AQP4 expression was associated with human glioma cancer development, suggesting that it triggered cell migration, invasion, and tumorigenesis [33,34]. Although study has not mentioned about AQP4 correlation with ovarian cancer, consistent with the previous results, we documented that *AQP4* mRNA expression in ovarian cancer was correlated with poor prognosis to OS in all ovarian cancer, especially with the serous ovarian cancer type. Similarly, high AQP4 level showed markedly unfavorable prognosis both in well and poorly differentiated cancer and early (I + II) and advanced stage (III + IV) ovarian cancer. Based on previous evidences as well as our results, we can acknowledge that *AQP0, AQP1*, and *AQP4* high mRNA expression were significantly associated with poor prognosis in ovarian cancer patients.

Kang et al. [35] reported that overexpression of AQP3 was associated with a worse prognosis in patients with HER2-positive early breast cancer. In a study of patients with cervical carcinomas, Shi et al. [36] observed that the expression of AQP3 was significantly increased in larger tumor size, advanced stage, deeper infiltration, and metastatic lymph nodes. Thus, they indicated that AQP3 might play an important role in carcinogenesis and tumor progression of cervical carcinomas [36]. To our knowledge, the study of Yang et al. [37] was the first to show that the expression of AQP3 was increased in epithelial ovarian cancer from laying hens, which suggested that AQP3 may be a potential biomarker to predict the development and progression of epithelial cell-derived ovarian carcinomas in chickens. However, there is no publication reporting on the prognostic significance of AQP3 in human ovarian cancer. Intriguingly, our study found that *AQP3* mRNA expression significantly associated with better prognosis in all ovarian cancer patients, both in endometrioid and serous types ovarian cancer patients. Moreover, overexpression of *AQP3* mRNA speculated favorable OS in poor differentiation and advanced clinical stage ovarian cancer patients. Similarly, Zhang et al. [38] reported that AQP5 played a key role in cervical cancer and was linked with a worse prognosis in patients with cervical cancer. Furthermore, Yang et al. [39] showed that the expression of AQP5 protein and mRNA was positively correlated with ascites amount and lymph node metastasis, and they speculated that AQP5 might play a critical role in tumorigenesis of epithelial ovarian tumors. However, the study about the prognostic role of AQP5 in ovarian cancer has not been studied yet. Our results showed that *AQP5* mRNA expression was significantly associated with better OS in all ovarian cancer patients and well differentiated ovarian cancer patients. Taken together, our results indicated that high level of AQP3 and AQP5 might favor clinical outcomes in ovarian cancer.

Ma et al. [40] reported that the level of AQP6 was obviously decreased in serous ovarian cancer compared with normal tissues. Nonetheless, whether or not AQP6 has a prognostic role in ovarian cancer remained elusive. In this report, *AQP6* mRNA expression found to be associated with better OS in all ovarian cancer patients, mainly in endometrioid cancer patients, as well as in all clinical stages and poorly differentiated ovarian cancer. In addition, Ma et al. [40] showed that there was no significantly statistical difference in AQP8 expression between benign and malignant epithelial ovarian tumors. However, they demonstrated that the AQP8 expression was increased in patients with large volume of malignant ascites, suggesting that the clinical significance of AQP8 needed further study. In the preent study, we reported for the first time that high *AQP8* mRNA expression was associated with favorable OS for all the ovarian cancer patients and advanced stage (III + IV) ovarian cancer. Therefore, high *AQP6* and *AQP8* mRNA expression may predict a favorable prognosis in ovarian cancer.

Different from other AQPs, there was no previous study about the role of AQP10 and AQP11 in human malignant tumors. In the present study, we found that high expression of *AQP10* mRNA predicted a better prognosis in all ovarian cancer patients mostly with serous subtypes, well-differentiated pathological grade (grade I) and advanced stage (III + IV) ovarian cancer. In addition, we observed a better OS with higher *AQP11* mRNA expression in all ovarian cancer patients mainly with serous subtypes, as well as in poorly differentiated (grade III) cancer patients. In general, our results showed that the presence of *AQP10* and *AQP11* mRNA linked with positive outcomes in ovarian cancer patients.

Large family members of AQPs and mutation of a tumor suppressor gene have been considered as an essential driver in cancer onset and progression. *TP53* gene transcription regulators are extensively studied and highly mutated in various aspects of ovarian cancer. It is frequently located in early events, generally with high-grade serous ovarian cancers [41]. Additionally, study has suggested that activated p53 could regulate the expression of AQPs through p38 MAPK pathway and mediate the cytotoxic effects [42,43]. Multiple evidences have revealed the correlation between p53 function and ovarian cancer stem cells, however, evidences about the prognostic association between TP53 status and AQP subtypes in ovarian cancer remains to be clarified. In our current analysis, we found that high level of *AQP1, AQP4*, and *AQP8* mRNA expression in TP53 mutation and *AQP1* mRNA expression in TP53 wild-type ovarian cancer patients were correlated with poor survival rate, indicating that mutation of *TP53* gene might regulate AQP1, AQP4, and AQP8 expression and participation in the development of ovarian caners.

Recently, AQPs were found to be permeable with arsenic and antimony compounds and took part in the detoxification pathway thereby conferring chemoresistance and sensitivity via transporting metalloids into cells [44–46]. It has been anticipated that finding AQP inhibitors of suitable affinity and specificity will generate highly acceptable pharmacological tools and contribute as a potential novel chemotherapeutic agent [47]. Xuejun et al. [48] suggested that AQPs were closely associated with other transmembrane transport channels, and thus played a vital role in cell apoptosis, drug metabolism, and chemosensitivity through water permeability regulation in ovarian cancer. In addition, they found different members of AQPs displayed various responses to chemotherapy in ovarian cancer cells. The relation between AQP signaling and carcinogenesis, as well as its cross-talk with multiple oncogenic signaling pathway implied that AQP signaling, mainly few AQP receptors might be a good candidate for drug target of ovarian cancer. In the present study, we observed that almost all studied AQPs submembers were correlated to the prognosis referring to Platin chemotherapeutic agent. In fact, *AQP0, AQP1*, and *AQP4* mRNA overexpression were associated with a poor prognosis with Platin therapy. However, *AQP3, AQP5, AQP6, AQP8, AQP10*, and *AQP11* high mRNA were associated with improved survival rate, implying that overexpression of these six AQP submembers increase drug sensitivity, probably through eliminating most infiltrative cells and increased intracellular accumulation of drugs which later permeated through special transmembrane transport system or extracellular hyperosmosis mechanism [44,48,49]. So far, the exact mechanism is unknown but abnormal expression of AQPs in hypertonic stress might have contributed to inhibit transmembrane transport of platinum ion in ovarian cancer cells [49]. In addition, confer to the Taxol-treated ovarian cancer patients, elevated *AQP4, AQP5, AQP8*, and *AQP10* mRNA expression illustrated no correlation, whereas increased expression of AQP0 and AQP1 revealed poor OS and increased expression of AQP3, AQP6, and AQP11 showed improved OS in ovarian cancer patients. Intriguingly, when concurrent (Platin + Taxol) treatment and prognostic significance were analyzed, the data showed that high expression of *AQP0* and *AQP1* mRNA were correlated to decrease survival rate and high expression of AQP3, AQP6, and AQP11 were correlated to good OS in ovarian cancer patients. In general, AQPs and its submembers might contribute to the overall survival, however, due to other independent function of regulatory transport mechanism of chemotherapeutic across cell membrane, it can be a significant therapeutic target in ovarian cancer patients, yet discrete significance of AQPs expression needs to be further explored in order to specify independent function.

## Conclusion

Based on the above-mentioned comprehensive survival analysis platforms of KM plotter, our results showed that six members of *AQPs* (*AQP3, AQP5, AQP6, AQP8, AQP10*, and *AQP11*) mRNA expression were significantly correlated to favorable OS in ovarian cancer patients, whereas *AQP0, AQP1*, and *AQP4* mRNA were associated with poor survival in ovarian cancer patients. In addition, high level of AQPs have been detected with essential prognostic effects in Platin, Taxol, and concurrent-based chemotherapy in ovarian cancer. We also observed that specific AQPs were associated with the pivotal role in the prognosis of early and advanced clinical stages, different pathological grade, and TP53 status in ovarian cancer patients. These results indicated that individual AQPs, except AQP2 and AQP9, were associated with unique prognostic significance and thus might act as new predictive prognostic indicators in ovarian cancer. Even though our findings were statistically significant, large family members of aquaporin genes need future studies to establish the regulatory pathway of specific AQPs in carcinogenesis, tumor progression and invasion of ovarian cancer. Current study provided new insights regarding the contribution of AQPs subtypes into ovarian cancer progression and might help to explore the further discovery of AQPs to contribute as an accurate and strong cancer prognostic predictor and develop as a potential drug therapeutic target.

## Abbreviations

AQP: aquaporin
HR: hazard ratio
KM plotter: Kaplan–Meier plotter
OS: overall survival

## References

[1] Jemal A. (2010) Cancer statistics, 2010. CA Cancer J. Clin. 60, 277–300 10.3322/caac.20073 20610543

[2] Vergote I. (2010) Neoadjuvant chemotherapy or primary surgery in stage IIIC or IV ovarian cancer. N. Engl. J. Med. 363, 943–953 10.1056/NEJMoa0908806 20818904

[3] Leung P.C. and Choi J.H. (2007) Endocrine signaling in ovarian surface epithelium and cancer. Hum. Reprod. Update 13, 143–162 10.1093/humupd/dml002 17071638

[4] Foley O.W., Rauh-Hain J.A. and del Carmen M.G. (2013) Recurrent epithelial ovarian cancer: an update on treatment. Oncology (Williston Park) 27, 288–294, 298 23781692

[5] Coleman M.P. (2011) Cancer survival in Australia, Canada, Denmark, Norway, Sweden, and the UK, 1995-2007 (the International Cancer Benchmarking Partnership): an analysis of population-based cancer registry data. Lancet 377, 127–138 10.1016/S0140-6736(10)62231-3 21183212 PMC3018568

[6] Rubenwolf P. (2015) Loss of AQP3 protein expression is associated with worse progression-free and cancer-specific survival in patients with muscle-invasive bladder cancer. World J. Urol. 33, 1959–1964 10.1007/s00345-015-1574-8 25939538

[7] Yasui M. (1999) Rapid gating and anion permeability of an intracellular aquaporin. Nature 402, 184–187 10.1038/46045 10647010

[8] Soria L.R. (2010) Aquaporin-8-facilitated mitochondrial ammonia transport. Biochem. Biophys. Res. Commun. 393, 217–221 10.1016/j.bbrc.2010.01.104 20132793

[9] Guo X. (2013) Prognostic value of combined aquaporin 3 and aquaporin 5 overexpression in hepatocellular carcinoma. Biomed. Res. Int. 2013, 206525 10.1155/2013/206525 24224160 PMC3810059

[10] Saito T. (2018) Proteomic analysis of AQP11-null kidney: proximal tubular type polycystic kidney disease. Biochem. Biophys. Rep. 13, 17–21 29204517 10.1016/j.bbrep.2017.11.003 PMC5709289

[11] Carbrey J.M. and Agre P. (2009) Discovery of the aquaporins and development of the field. Handb. Exp. Pharmacol. 3–28 10.1007/978-3-540-79885-9_1 19096770

[12] Saadoun S. (2005) Impairment of angiogenesis and cell migration by targeted aquaporin-1 gene disruption. Nature 434, 786–792 10.1038/nature03460 15815633

[13] Ishibashi K., Hara S. and Kondo S. (2009) Aquaporin water channels in mammals. Clin. Exp. Nephrol. 13, 107–117 10.1007/s10157-008-0118-6 19085041

[14] King L.S. and Agre P. (1996) Pathophysiology of the aquaporin water channels. Annu. Rev. Physiol. 58, 619–648 10.1146/annurev.ph.58.030196.003155 8815812

[15] Verkman A.S. (2002) Physiological importance of aquaporin water channels. Ann. Med. 34, 192–200 10.1080/ann.34.3.192.200 12173689

[16] Gyorffy B. (2010) An online survival analysis tool to rapidly assess the effect of 22,277 genes on breast cancer prognosis using microarray data of 1,809 patients. Breast Cancer Res. Treat. 123, 725–731 10.1007/s10549-009-0674-9 20020197

[17] Tilghman S.L. (2013) Proteomic signatures of acquired letrozole resistance in breast cancer: suppressed estrogen signaling and increased cell motility and invasiveness. Mol. Cell Proteomics 12, 2440–2455 10.1074/mcp.M112.023861 23704778 PMC3769322

[18] Ivanova L. (2015) Prognostic relevance of carbonic anhydrase IX expression is distinct in various subtypes of breast cancer and its silencing suppresses self-renewal capacity of breast cancer cells. Cancer Chemother. Pharmacol. 75, 235–246 10.1007/s00280-014-2635-1 25422154

[19] Gayarre J. (2016) The NER-related gene GTF2H5 predicts survival in high-grade serous ovarian cancer patients. J. Gynecol. Oncol. 27, e7 10.3802/jgo.2016.27.e7 26463438 PMC4695457

[20] Gyorffy B., Lanczky A. and Szallasi Z. (2012) Implementing an online tool for genome-wide validation of survival-associated biomarkers in ovarian-cancer using microarray data from 1287 patients. Endocr. Relat. Cancer 19, 197–208 10.1530/ERC-11-0329 22277193

[21] Gyorffy B. (2013) Online survival analysis software to assess the prognostic value of biomarkers using transcriptomic data in non-small-cell lung cancer. PLoS ONE 8, e82241 10.1371/journal.pone.0082241 24367507 PMC3867325

[22] Xia P. and Xu X.Y. (2016) Prognostic significance of CD44 in human colon cancer and gastric cancer: evidence from bioinformatic analyses. Oncotarget 7, 45538–45546 10.18632/oncotarget.9998 27323782 PMC5216740

[23] Kishore B.K. (2000) Expression of renal aquaporins 1, 2, and 3 in a rat model of cisplatin-induced polyuria. Kidney Int. 58, 701–711 10.1046/j.1523-1755.2000.00216.x 10916093

[24] Huang D. (2017) AQP9-induced cell cycle arrest is associated with RAS activation and improves chemotherapy treatment efficacy in colorectal cancer. Cell Death Dis. 8, e2894 10.1038/cddis.2017.282 28640255 PMC5520935

[25] Tan G., Sun S.Q. and Yuan D.L. (2008) Expression of the water channel protein aquaporin-9 in human astrocytic tumours: correlation with pathological grade. J. Int. Med. Res. 36, 777–782 10.1177/147323000803600420 18652774

[26] Yang J.H. (2011) Expression of aquaglyceroporins in epithelial ovarian tumours and their clinical significance. J. Int. Med. Res. 39, 702–711 10.1177/147323001103900302 21819701

[27] Imredi E. (2016) Aquaporin 1 protein expression is associated with BRAF V600 mutation and adverse prognosis in cutaneous melanoma. Melanoma Res. 26, 254–260 10.1097/CMR.0000000000000243 26848795

[28] Lehnerdt G.F. (2015) AQP1, AQP5, Bcl-2 and p16 in pharyngeal squamous cell carcinoma. J. Laryngol. Otol. 129, 580–586 10.1017/S002221511500119X 26074259

[29] Liu J., Zhang W.Y. and Ding D.G. (2015) Expression of aquaporin 1 in bladder uroepithelial cell carcinoma and its relevance to recurrence. Asian Pac. J. Cancer Prev. 16, 3973–3976 10.7314/APJCP.2015.16.9.3973 25987071

[30] Qin F. (2016) Expression of aquaporin1, a water channel protein, in cytoplasm is negatively correlated with prognosis of breast cancer patients. Oncotarget 7, 8143–8154 10.18632/oncotarget.6994 26812884 PMC4884982

[31] Yang J.H. (2006) The influence of aquaporin-1 and microvessel density on ovarian carcinogenesis and ascites formation. Int. J. Gynecol. Cancer 16, 400–405 10.1111/j.1525-1438.2006.00476.x 16515633

[32] Sun X.L. (2007) Aquaporin 4 regulates the effects of ovarian hormones on monoamine neurotransmission. Biochem. Biophys. Res. Commun. 353, 457–462 10.1016/j.bbrc.2006.12.040 17196551

[33] Saadoun S. (2002) Aquaporin-4 expression is increased in oedematous human brain tumours. J. Neurol. Neurosurg. Psychiatry 72, 262–265 10.1136/jnnp.72.2.262 11796780 PMC1737753

[34] Ding T. (2011) Role of aquaporin-4 in the regulation of migration and invasion of human glioma cells. Int. J. Oncol. 38, 1521–1531 21424125 10.3892/ijo.2011.983

[35] Kang S. (2015) Aquaporin 3 expression predicts survival in patients with HER2-positive early breast cancer. Anticancer Res. 35, 2775–2782 25964557

[36] Shi Y.H. (2012) Significance and expression of aquaporin 1, 3, 8 in cervical carcinoma in Xinjiang Uygur women of China. Asian Pac. J. Cancer Prev. 13, 1971–1975 10.7314/APJCP.2012.13.5.1971 22901156

[37] Yang C. (2016) Aquaporin 3 is regulated by estrogen in the chicken oviduct and is involved in progression of epithelial cell-derived ovarian carcinomas. Domest. Anim. Endocrinol. 55, 97–106 10.1016/j.domaniend.2015.12.003 26808975

[38] Zhang T. (2012) Overexpression of AQP5 in cervical cancer: correlation with clinicopathological features and prognosis. Med. Oncol. 29, 1998–2004 10.1007/s12032-011-0095-6 22048942

[39] Yang J.H. (2006) Expression and localization of aquaporin-5 in the epithelial ovarian tumors. Gynecol. Oncol. 100, 294–299 10.1016/j.ygyno.2005.08.054 16242760

[40] Ma J. (2016) Expression of AQP6 and AQP8 in epithelial ovarian tumor. J. Mol. Histol. 47, 129–134 10.1007/s10735-016-9657-4 26779650

[41] Lee Y. (2007) A candidate precursor to serous carcinoma that originates in the distal fallopian tube. J. Pathol. 211, 26–35 10.1002/path.2091 17117391

[42] Yan J.H. (2012) p53-induced uncoupling expression of aquaporin-4 and inwardly rectifying K+ 4.1 channels in cytotoxic edema after subarachnoid hemorrhage. CNS Neurosci. Ther. 18, 334–342 10.1111/j.1755-5949.2012.00299.x 22420318 PMC6493666

[43] Zheng X. and Chen X. (2001) Aquaporin 3, a glycerol and water transporter, is regulated by p73 of the p53 family. FEBS Lett. 489, 4–7 10.1016/S0014-5793(00)02437-6 11231003

[44] Bhattacharjee H., Rosen B.P. and Mukhopadhyay R. (2009) Aquaglyceroporins and metalloid transport: implications in human diseases. Handb. Exp. Pharmacol., 309–325 10.1007/978-3-540-79885-9_16 19096785 PMC2729095

[45] Leung J. (2007) Relationship of expression of aquaglyceroporin 9 with arsenic uptake and sensitivity in leukemia cells. Blood 109, 740–746 10.1182/blood-2006-04-019588 16968895

[46] Naranmandura H. (2009) Evidence for toxicity differences between inorganic arsenite and thioarsenicals in human bladder cancer cells. Toxicol. Appl. Pharmacol. 238, 133–140 10.1016/j.taap.2009.05.006 19442679

[47] Song J. (2014) Parasite aquaporins: current developments in drug facilitation and resistance. Biochim. Biophys. Acta 1840, 1566–1573 10.1016/j.bbagen.2013.10.014 24140393

[48] Xuejun C. (2014) Effects of aquaporins on chemosensitivity to cisplatin in ovarian cancer cells. Arch. Gynecol. Obstet. 290, 525–532 10.1007/s00404-014-3216-6 24695904

[49] Chen X. (2015) Hyperosmotic stress induces cisplatin sensitivity in ovarian cancer cells by stimulating aquaporin-5 expression. Exp. Ther. Med. 10, 2055–2062 10.3892/etm.2015.2833 26668595 PMC4665691

